# Epidemiology of Breakthrough Varicella after the Implementation of a Universal Varicella Vaccination Program in Taiwan, 2004–2014

**DOI:** 10.1038/s41598-018-35451-y

**Published:** 2018-11-21

**Authors:** Hao-Yuan Cheng, Luan-Yin Chang, Chun-Yi Lu, Li-Min Huang

**Affiliations:** 1 Taiwan Centers for Disease Control, Taipei, Taiwan; 2 Department of Pediatrics, National Taiwan University Children’s Hospital, National Taiwan University College of Medicine, Taipei, Taiwan

## Abstract

National one-dose varicella vaccination at 12 months of age was implemented in Taiwan since 2004.Our study aimed to evaluate breakthrough varicella (BV) in post-vaccine era and its associated risk factors. We retrospectively identified children vaccinated against varicella between 12–23 months of age during 2004–2008. Their vaccination information was extracted from the national vaccination registry system and linked to the 2004–2014 National Health Insurance database. BV was defined as a diagnosis of varicella (ICD-9-CM codes 052 and 052.0–052.9) beyond 42 days post-vaccination. Multiple Cox regression model was used to identify risk factors for BV. Among 932,874 enrolled vaccinees, 26,446 (2.8%) had BV and 219 (0.024%) required hospitalization over the study period. Varicella incidence declined from 4.71 per 1000 person-year (PY) in 2004 to 0.81/1000 PY in 2014. BV incidence decreased from 3.90/1000 PY at first year to 1.94/1000 PY at 11th year after vaccination. Females had a lower risk for BV than males (hazard ratio [HR] 0.85, 95% CI, 0.83–0.87); Varivax^®^ recipients had a lower risk for BV than Varilrix^®^ recipients (HR 0.75, 95% CI, 0.72–0.78). Our study showed the incidence of varicella, BV and varicella-related hospitalizations in Taiwan were kept low in post-vaccine era.

## Introduction

Varicella vaccine was first developed in 1974 and is currently the most effective method to prevent varicella in children. A meta-analysis in 2016 estimated the pooled vaccine effectiveness of one-dose varicella vaccines in routine clinical practice at 81% (95% confidence interval [CI] 78–84%), with effectiveness of two doses estimated at 92% (95% CI 88–95%)^1^. Live attenuated varicella vaccine was licensed in Taiwan in 1997, and Taipei City and Taichung City began providing one free dose of varicella vaccine to one-year-old children in 1998 and 1999, respectively. Universal varicella vaccination (UVV) with one dose at 12 months of age was not implemented at the national level until 2004. Two brands of OKA-strain varicella vaccines were used in Taiwan: Varivax^®^ (Merck & Co., Inc., USA) and Varilrix^®^ (GlaxoSmithKline plc, UK). After the introduction of UVV through the nationally funded program in 2004, both vaccines continued to be used, but were primarily based on government tender awards, which were divided into approximately equal time periods amongst Varivax^®^ (01/2004–06/2006, 01/2009–12/2011) and Varilrix^®^ (07/2006–12/2008, 01/2012–12/2014). An evaluation conducted shortly after the implementation of the UVV program showed that varicella vaccines had an effectiveness of 85.4% against hospitalized varicella and 82% against all clinical varicella^2^. The crude varicella incidence also decreased from 5.68 in 2003 (pre-vaccination era) to 2.23 per 1,000 persons at five years after implementation of UVV in 2007^3^.

Although varicella declined in the first four years of routine vaccine use and vaccine coverage was generally over 95%, varicella outbreaks still occurred in some schools each year in Taiwan. In addition, even in the vaccinated population, breakthrough varicella (BV) was reported. Similar concerns about vaccine effectiveness in routine practice, the possibility of decreasing vaccine-induced immunity, lead to the implementation of a second routine dose of varicella vaccine in the US^4,5^. Some researchers have questioned whether vaccine-induced immunity can persist for a long period of time without natural boosting from circulating varicella-zoster virus in the community^5^.

In Taiwan, a sero-epidemiological survey in school children in 2013 reported that only approximately 64.1% of children in primary school (aged 7–12 years) remained seropositive for varicella-zoster virus using an indirect chemiluminescence immunoassay (Liaison, DiaSorin, Italy)^6^. However, this result could be due to the low sensitivity of commercial serological assays in picking up vaccine-induced immunity^7,8^. No robust evaluation of the effectiveness of the universal varicella vaccination program and the incidence of BV has yet been performed since the start of UVV.

Therefore, the objective of the present study was to evaluate the impact of the universal one-dose varicella vaccination program, to describe the epidemiology and temporal pattern of BV after UVV, and to identify corresponding risk factors.

## Results

### Vaccine impact and effectiveness

**Figure 1:**
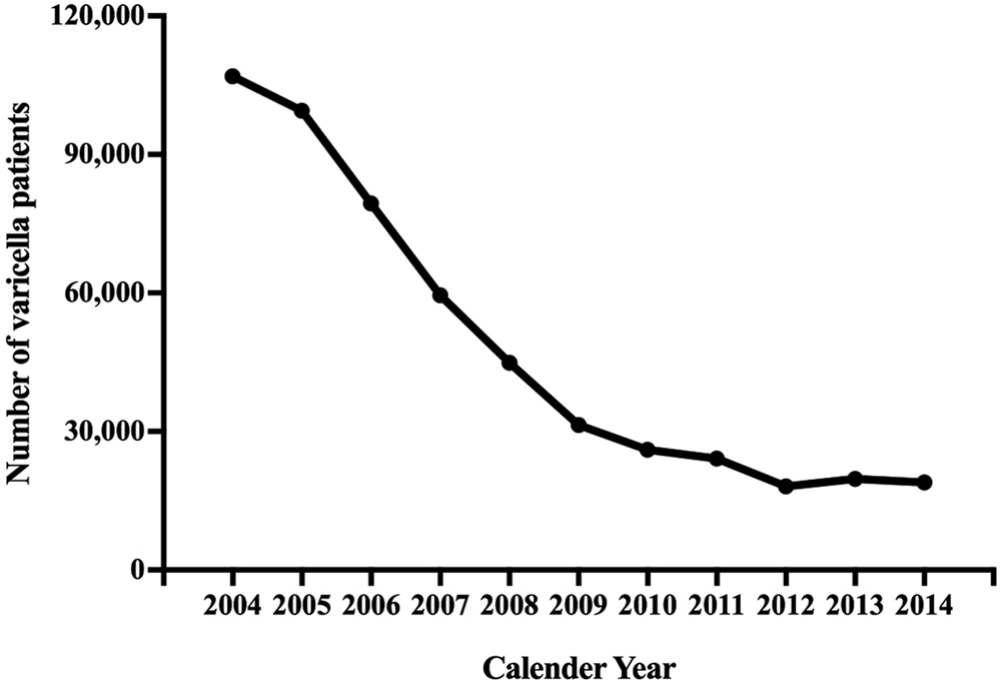
Temporal trends of the number of annual total varicella patients in Taiwan, 2004–2014.

The annual number of varicella patients in all age groups recorded in the National Health Insurance (NHI) database decreased from 106,913 in 2004 to 18,960 in 2014 (Fig. 1), which constitutes a reduction of more than 80% of all varicella patients during the 11-year routine varicella vaccination program. The number of varicella cases dropped more rapidly shortly after the start of UVV, and the decline slowed down after 2012. The crude varicella incidence also steadily declined from 4.71 per 1,000 person-year (PY) in 2004 to 0.81/1000 PY in 2014. Overall direct vaccine effectiveness was estimated to range from 82.0% (95% CI 81.3–82.6%) to 93.1% (95% CI 92.7–93.4%) in the birth cohorts from 2003–2006 (Table 1).

**Table 1:** Vaccine effectiveness estimated by birth cohorts using the screening method, 2004–2014.

Birth cohort	Years of Follow up	BV cases	Vaccinated children	VE	95% CI
2003	11	7,659	208,437	82.0%	81.3–82.6%
2004	10	5,571	205,534	88.5%	88.0–89.0%
2005	9	4,211	198,198	93.1%	92.7–93.4%
2006	8	3,289	196,284	88.9%	88.4–89.5%

Each cohort was followed until the end of 2014, so the 2003 cohort had up to 11 years of follow-up, whereas the 2006 cohort had only 8 years of follow-up. The 2007 birth cohort is not shown because it was not a full cohort (some of those born in 2007 may have been vaccinated in 2009, and hence not captured in this study).

### Breakthrough varicella

**Figure 2:**
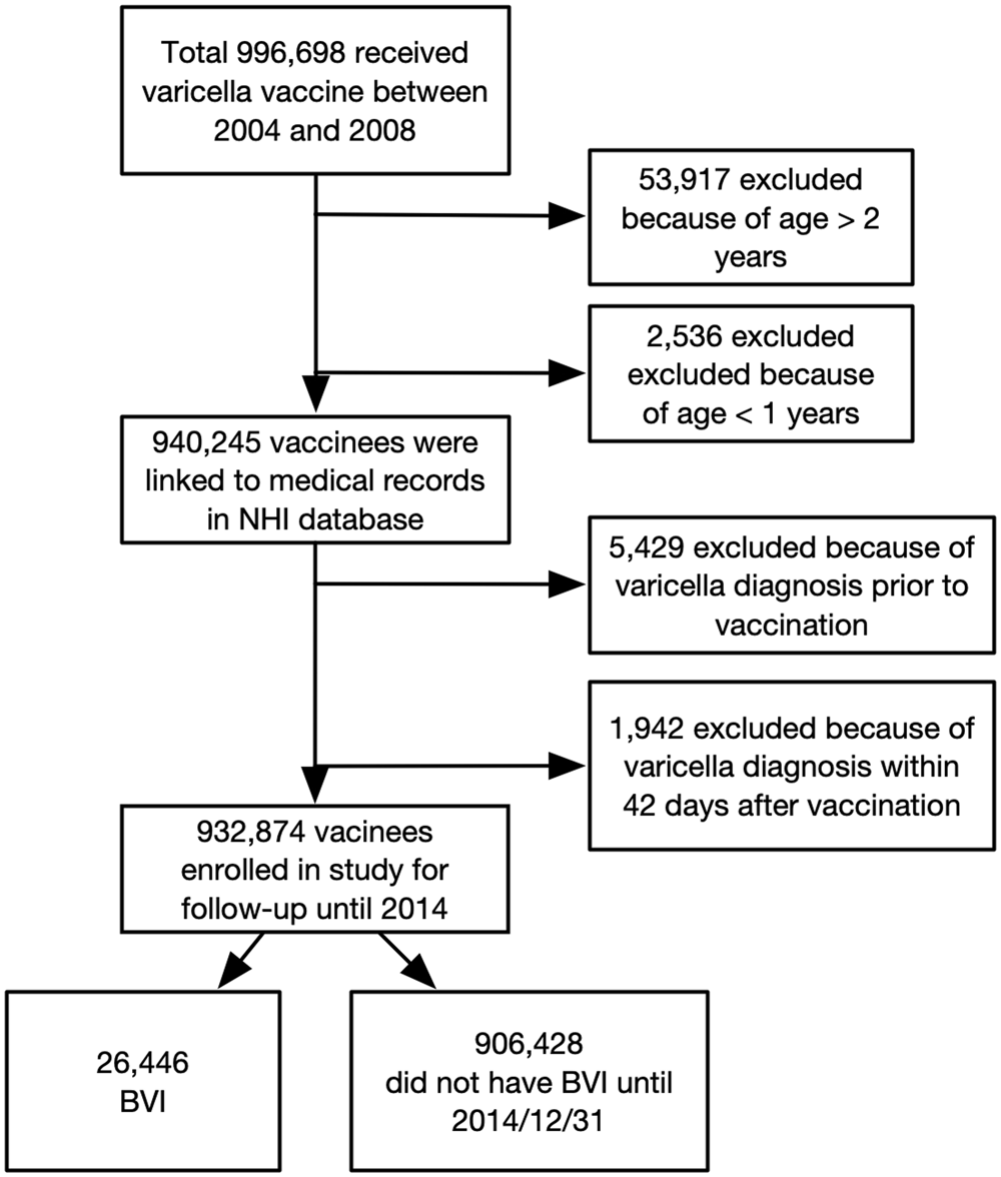
Case enrollment flowchart. Each cohort was followed until the end of 2014. The 2003 cohort had up to 11 years of follow-up, whereas the 2007 cohort had only seven years of follow-up.

A total of 996,698 children received varicella vaccine between 2004 and 2008 during the universal vaccine program. Among these vaccinees, 53,917 children were older than two years, and 2,536 children were younger than one year at vaccination and were excluded. After further excluding those who had been diagnosed with varicella <42 days after vaccination, 932,874 vaccinees 12–23 months of age were enrolled in the analysis (Fig. 2). Mean duration of follow-up time was 8.45 years.

BV occurred among 26,446 (2.8%) vaccinees and 219 (0.024%) required hospitalization (Table 2). Median ages at vaccination and at BV were 1.05 (range 1–1.97) and 5 (range 1–12) years, respectively. The median interval between vaccination and BV was 3.90 years (range 43.8 days–10.81 years). The male to female ratio was 1.28, and the proportion of BV in males was slightly higher than in females (3.1% vs. 2.6%, p < 0.001). The proportion of BV in those from different geographic regions was similar.

**Table 2:** Basic characteristics of children vaccinated with varicella vaccine at age 12–23 months between 2004–2008 (vaccinees) and their breakthrough varicella (BV).

	All vaccinees (n = 932,874)	Vaccinees with BV (n = 26,446)
Age at vaccination (years)	1.04 (1–1.97)	1.05 (1–1.97)
Age at BV (years)		5 (1–12)
Sex*		
Male	487,754	14,869 (3.1%)
Female	445,113	11,577 (2.6%)
Vaccine brand*		
Varilrix^®^	299,882	8893 (3.0%)
Varivax^®^	544,909	15,357 (2.8%)
Others/unknown	88,083	2196 (2.5%)
**Reside in counties with universal varicella vaccination implemented before the national program in 2004**		
Yes	712,991	20,280 (2.8%)
No	219,883	6,166 (2.8%)

*p value < 0.05. All expressed as median (range) or n (%).

**Figure 3:**
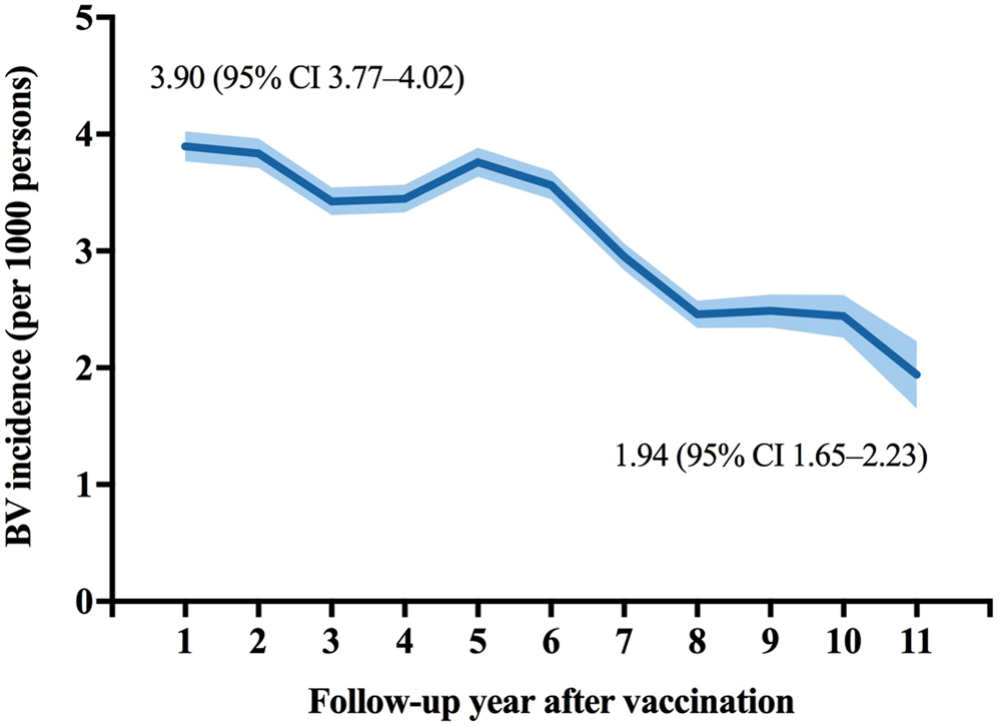
Annual BV incidence in all varicella vaccinees by follow-up years, 2004–2014. Solid lines represent point estimates, and shadowed lines represent 95% confidence intervals.

**Figure 4:**
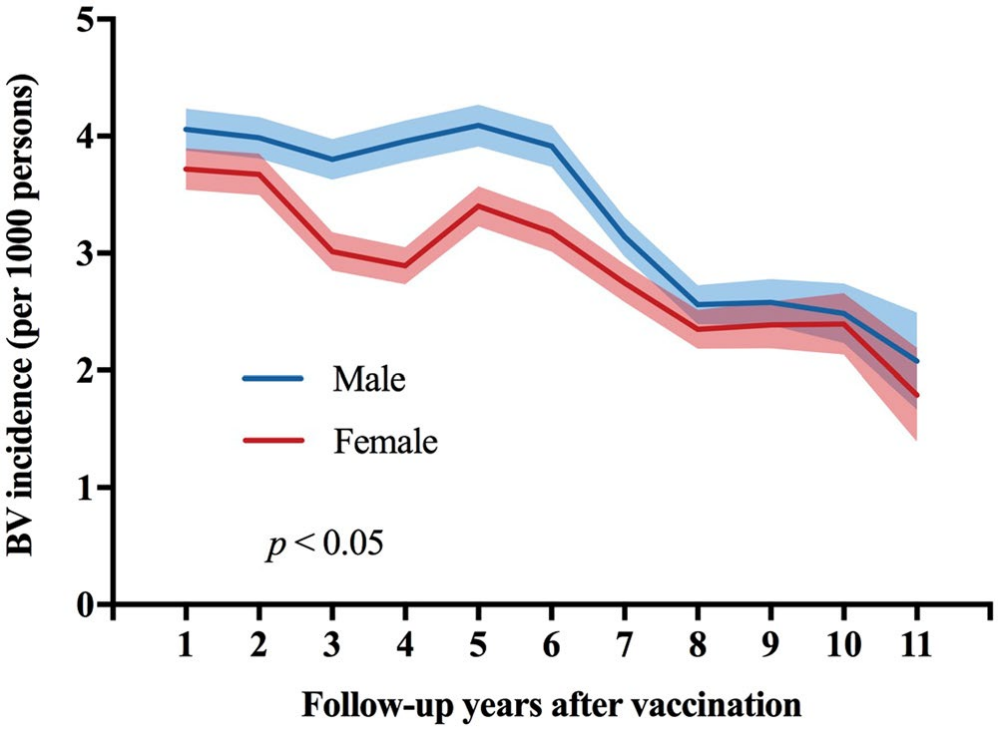
Annual BV incidence in all varicella vaccinees by follow-up years and sex, 2004–2014. Solid lines represent point estimates, and shadowed lines represent 95% confidence intervals. The p-value refers to the statistical significance of the overall effect over the full-time period based on the Cox proportional hazards model.

Using the life table method, we found that the annual BV incidence decreased after vaccination, from 3.90/1000 PY one year following vaccination to 1.94/1000 PY 11 years after vaccination (Fig. 3). BV incidence among males was steadily higher than females throughout the entire follow-up period. In males, BV incidence decreased from 4.06/1000 PY one year after vaccination to 2.08/1000 PY 11 years after vaccination. In females, it decreased from 3.71/1000 PY to 1.79/1000 PY over the same time period (Fig. 4).

**Figure 5:**
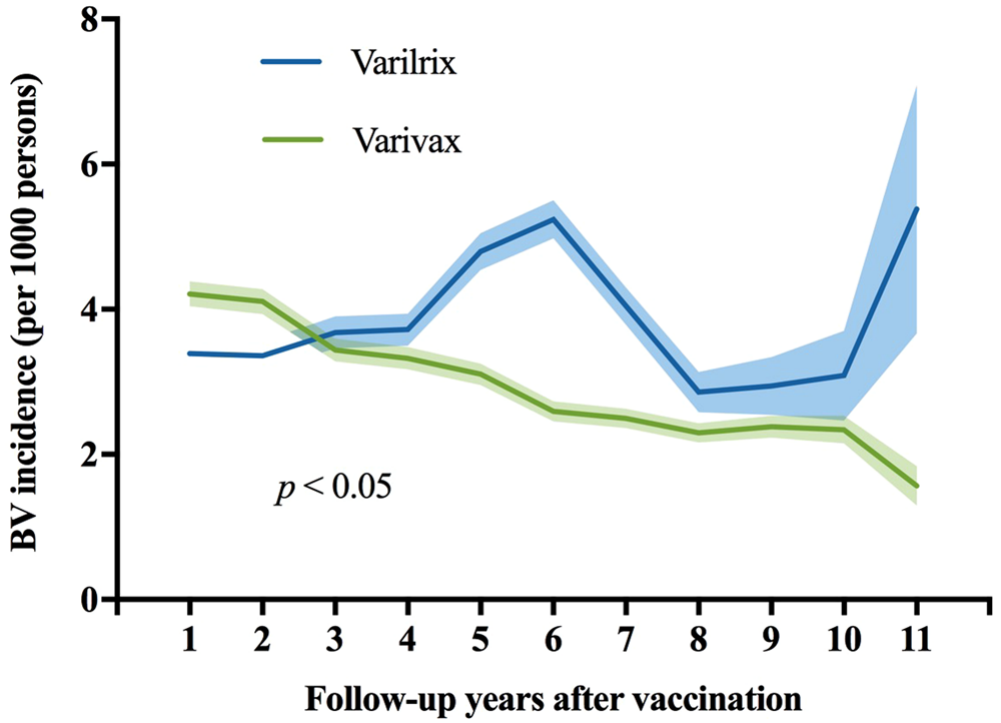
Annual BV incidence in all varicella vaccinees by follow-up years and manufacturers, 2004–2014. Solid lines represent point estimates, and shadowed lines represent 95% confidence intervals. The p-value refers to the statistical significance of the overall effect over the full-time period based on the Cox Proportional Hazards model.

When vaccines of different manufacturers were considered, the overall BV rate over the study period was 2.82% for Varivax^®^ and 2.97% for Varilrix^®^ (Table 2). The BV incidence following receipt of Varivax^®^ consistently decreased from 4.21/1000 PY at 1 year, 2.58/1000 PY at 6 years and 1.57/1000 at 11 years after vaccination. On the other hand, while the BV incidence at 1 year after vaccination among recipients of Varilrix^®^ was 3.66/1000 PY, two peaks were observed at 6 and 11 years after vaccination (5.24/1000 PY and 5.38/1000 PY, respectively), with a sharp increase beyond year 10 (Fig. 5). In the sub-group analysis categorized by vaccination cohort from 2004 to 2008, the temporal patterns of the incidence of BV were also different in recipients of vaccines from different manufacturers (see Supplement Fig. S1).

Using the Cox proportional hazards regression model, the results were stratified by geographic region and calendar year at vaccination to ensure that the comparison was among those who received vaccination in the same (geographic) place and during the same year to minimize the effect of exposure (either through boosting or herd immunity effects) to wild varicella virus circulation in the early years of the program, and adjust for the higher vaccination coverage pre-2004 in early introduction areas. Even after this adjustment, females had a lower risk for BV as compared to males (hazard ratio [HR] 0.85, 95% confidence interval [CI] 0.83–0.87), while recipients of Varivax^®^ also had a significantly lower risk for BV than that for recipients of Varilrix^®^ (HR 0.75, 95% CI 0.72–0.78) (Table 3).

**Table 3:** Cox proportional hazard regression model* for BV risk factors.

	Hazard ratio	95% CI
Female vs. male	0.85	0.83–0.87
Varivax^®^ vs. Varilrix^®^	0.75	0.72–0.78
Age at vaccination (month)	1.13	1.09–1.17

*Stratified by county of residence and calendar year at vaccination.

## Discussion

This study of close to one million children revealed a high level of effectiveness of a universal one-dose varicella program in Taiwan, based on the significant decrease (82.3%) in the overall number of varicella cases, reduction in varicella incidence (4.71/1000PY in 2004 to 0.81/1000 PY in 2014) and the limited number of varicella-related hospitalizations (<0.03%) reported over the first 11 years, together with low overall levels of BV incidence (2.8%) in Taiwan. In addition, there appeared to be no decrease of vaccine effectiveness up to 11 years after a single dose of vaccine. However, higher BV incidence was noted in males compared to females (3.1% and 2.6% over 11 years), and among recipients of Varilrix^®^ compared to recipients of Varivax^®^ (3.0% and 2.8% over 11 years).

The overall proportion of BV in varicella vaccinees was low even 11 years after the implementation of the universal vaccination program, and annual BV incidence in follow-up periods decreased from 3.90 to 1.94/1000 PY from one year to 11 years of follow-up. The cumulative BV incidence in children in Taiwan was similar to some previous studies in Taiwan and the US, but much lower than a few others in the US^2,4,9,10^. There are two possible explanations for the relatively lower incidence in this study. First, the varicella vaccine coverage rate in Taiwan rose rapidly, from 87% in 2004 to 95% by 2006, and remained between 95–99% between 2007 and 2014. In contrast, the US varicella vaccine programme did not reach 50% vaccine coverage until 5 years after UVV began, and only reached 90% coverage around the time of second dose introduction in 2005, ten years after the start of UVV. The higher vaccine coverage in Taiwan may contribute to better herd immunity and lower overall rates of virus circulation, and thus further reduce BV events. Second, this study used the NHI database to trace the outpatient and inpatient medical records of children with varicella, meaning that a study subject may not be detected as a BV patient if his or her varicella was not severe enough to warrant medical assistance. Therefore, the decreasing trends of BV identified in our study may only represent the decrease of patients with moderate to severe varicella disease, and thus differ from the pattern demonstrated in other studies using active surveillance data, which may capture more patients with mild disease^4,11^.

These results suggest that a one-dose varicella program may have been more effective than expected in Taiwan. Although mild varicella may still be infectious and result in further varicella outbreaks in populous settings, such as schools or households^5,12^, the direct medical cost related to mild varicella would be limited, and alone does not warrant a second-dose of varicella vaccine given that the total number of varicella infections requiring medical attention has largely decreased. However, it is important to continue to monitor the evolution of the disease in the community, as well as to characterize the impact that even mild disease can have on broader societal issues and costs, such as missed school days due to illness, parental lost work time, cost of outbreak investigation, and reduced confidence in the vaccine.

Interestingly, we found that the BV incidence in females was lower than in males. This finding was compatible with the observation made in another varicella vaccine study in Taiwan in 2011^2^. A similar sex effect was observed with measles vaccine, suggesting that women had a better immunological response to a live attenuated vaccine, and this might lead to better immunogenicity and longer protection^13,14^. Biologically, this phenomenon could be explained by the effect of sex hormones on immune response. Testosterone was demonstrated to suppress interferon (IFN) γ and interleukin 4 secretion in T cells, while estrogen could improve T-helper (Th) Th1 responses at lower doses and enhance Th2 responses and antibody production at higher concentrations^15,16^. However, calculation of the overall vaccine effectiveness for particular subgroups (i.e., specifically for males or females, or Varivax^®^ or Varilrix^®^) usually requires estimates of the relative risk of varicella disease in a comparable unvaccinated population, such as a historical control group, that would allow estimation of both direct and indirect vaccine effects^17^. In this case, the estimation of indirect effects was challenging because of the complications in accounting for externalities and confounders, for example, the herd immunity provided for all subgroups, especially when the likelihood of exposure to varicella was greatly mitigated through overall reductions in virus circulation resulting from high vaccine coverage in Taiwan. Therefore, the method used in this study and the findings could be regarded as another evidence of better vaccine protection in females. In this study, although the temporal pattern of BV incidence was similar both in females and males, the incidence in males was consistently higher than in females throughout the entire follow-up period, raising questions about why this phenomenon is observed. However, with increasing time since vaccination, breakthrough rates for both males and females converged and thus we did not see statistically significant differences in the last 5 years of follow-up.

The other difference in vaccine effectiveness found in this study concerned the vaccine received. Because both Varilrix^®^ and Varivax^®^ were used in Taiwan between 2004 and 2014 (the study period) and vaccine coverage was high, brand-specific vaccine effectiveness estimates might be influenced by the herd immunity impact of the other vaccine in terms of reductions in overall virus circulation. Annual BV incidence after vaccination was thus considered as a surrogate to make a comparison. Recipients of Varilrix^®^ exhibited an increase in BV events between the fifth and sixth year after vaccination, and a second increase was observed in the 10^th^ to 11^th^ year of follow-up. In contrast, a similar pattern was not found among recipients of Varivax^®^. Although both Varilrix^®^ and Varivax^®^ use strains of the Oka virus, one study using the gpELISA assay to assess the immunogenicity of Varilrix^®^ and Varivax^®^ demonstrated that Varivax^®^ had better immunogenicity^18^. Others have reported that the clinical trial and effectiveness data for Varilrix^®^ are similar to the results reported for Varivax^®^, although a recent clinical study showed lower effectiveness of a single dose of Varilrix^®^ (65.4%, 95% CI 57.2–72.1%) than had previously been reported^19–21^. Additionally, researchers in Germany reported possible differences in vaccine effectiveness in 2008–2009^22^. It is worth noting that those studies were performed in outbreak settings where higher virus exposure might exaggerate the difference in vaccine effectiveness^23^. Taken together, these points may suggest that different manufacturing processes still contribute to slight differences between two different vaccine types during long-term follow-up or during more stressful setting such as outbreaks. One meta-analysis performed in 2016 showed the pooled one-dose vaccine effectiveness was similar between different types of vaccine, but most included studies did not consider the effects of length of time since vaccination, which can influence the risk for varicella infection over time^1^. Although the point estimate of pooled vaccine effectiveness in that meta-analysis of Varivax^®^ was higher than Varilrix^®^, the statistically insignificant difference could have been due to the relatively smaller sample size for Varilrix^®^, or to the failure to account for time since vaccination. A subsequent meta-regression analysis of breakthrough rates following a single dose of varicella vaccination did find that Varilrix^®^ had a statistically significant higher rate of BV^24^. Our large-scale study provides further evidence of a (small) difference in effectiveness of the two vaccines, based on outcomes in the general population in Taiwan. Although the NHI database used to retrieve BV records may only cover those who sought medical care for varicella, the findings from this large study showed that Varivax^®^ resulted in a consistent decrease in medically attended BV over 11 years of follow-up, whereas medically attended BV incidence after Varilrix^®^ was more variable and statistically higher overall comparing the similar pattern after 8 years of follow-up in the male-female comparison. The cumulative incidence in Varilrix^®^ recipients was still significantly higher than in Varivax^®^ recipients given slightly higher BV incidence in Varivax^®^ recipients during the early years of UVV.

Although the results from the Cox proportional hazards regression model demonstrate that the relative risk of BV is 85% in females compared to males, and 75% in recipients of a single dose of Varivax^®^ compared to Varilrix^®^, the absolute level of BV incidence (2.8%) has been reduced to a relatively low level in Taiwan after 11 years of universal varicella vaccination. These data suggest that although the effect size might be small, protection against any form of varicella requiring medical care persists longer and better than anticipated. Clinical trials and real world effectiveness studies in other settings have shown that a second dose of varicella vaccine can provide further reductions in varicella disease, as well as preventing outbreaks^25–28^. Mathematical models have been utilized to demonstrate that a second dose of varicella could improve vaccine effectiveness from 78–94.4% to over 95%^28–32^, but the cost-effectiveness of a universal second-dose program in Taiwan would have to be considered given the already low rates of BV and the low rates of hospitalization. Such evaluations could also examine the broader societal impact of BV when milder cases, which might result in outbreaks, school absence and parental work loss, are prevented. In that we way we could have a more comprehensive view on the potential mitigated risks for those risk groups we have shown in this study.

Limitations of this study are primarily related to reliance on the NHI database, which only tracks resource utilization of those who seek medical attention, meaning that those with mild varicella who do not seek medical attention may not be captured. Information on reason for hospitalization or severity of varicella disease was not available for hospitalized cases from the NHI database. Furthermore, the NHI database is reliant on correct ICD-9 coding, and its validity is dependent on the assumption that the clinical manifestations of varicella and its ICD-9 coding are both very specific. The limitation may have been be mitigated due to the ease of universal access to healthcare in Taiwan, which suggests people with milder illnesses still tend to visit the doctor. The completeness of the data in National Immunization Information System (NIIS) could have constituted a further possible limitation in the study. However, given that the overall varicella vaccination coverage amongst children 12–23 months reached 95% by 2006, this seems unlikely to have had a significant impact on the outcome of the study. The final limitation that must be pointed out is that this retrospective study was based on the national health insurance database and vaccination registry database, and no clinical information from the medical records were retrieved in the databases; consequently, clinical course of the medical records was not reviewed for validation, nor to further explore the reasons behind hospitalizations. Further clinical studies focused on the validation or the complications of varicella in the post-vaccine era could be helpful.

## Conclusion

In summary, the effectiveness of the one-dose universal varicella vaccination program in Taiwan was significant in reducing both BV and varicella-related hospitalizations during the 11 years of the universal varicella vaccination program. A second dose of varicella vaccine might further reduce BV, but a thorough cost-effectiveness study from a broad societal perspective is recommended to justify its implementation.

## Methods

### Study design and population

We conducted a retrospective cohort study, identifying children who received varicella vaccine between 12 and 23 months of age during 2004–2008 from NIIS in Taiwan.

NIIS was established as the national vaccination registry system in the early 1990s. Every time a child receives a vaccination, health care workers send basic demographic characteristics and information about the vaccine used (i.e., the dose of vaccine and its manufacturer) to the database. Through this system, we identified all varicella vaccinees during 2004–2008 and collected their personal identification number (PIN), sex, date of birth, date of vaccination, and vaccine manufacturer. In order to minimize the effect of age at vaccination, we only enrolled those aged 12–23 months at vaccination in the analysis for BV. Those children with missing vaccination record for varicella vaccine were considered as non-vaccinated. After establishing our cohort, we then followed their BV status using the NHI database.

The NHI database in Taiwan contains all of the health care claims data from over 95% of the hospitals and clinics in Taiwan, including records for both outpatient and inpatient departments^4^. By using the PIN, the vaccinated cohort was linked to the 2004–2014 NHI database. We extracted the claims data of those who had any varicella-zoster virus infection using the *International Classification of Diseases, Ninth Revision, Clinical Modification*, ICD-9-CM code 052 and 052.0–52.9 for both vaccinated and unvaccinated people from the NHI database, including the date of diagnosis.

An event was classified as breakthrough varicella if a varicella case occurred ≥42 days post-vaccination^33^.

### Ethical approval

National Taiwan University Hospital’s institutional review board approved all study protocols.

We confirm that all research was performed in accordance with relevant guidelines/regulations. Because only health databases were used, there was no need to get individual informed consent for this study.

### Statistical analysis

VE was calculated by using the birth cohorts from 2003–2006, corresponding to vaccinations administered between 12–23 months of age in 2004–2008. The number of vaccinees in each birth cohort was counted according to the NIIS database. VE was defined as the relative reduction in the incidence rate of varicella in vaccinated, compared to unvaccinated, subjects from the same birth cohort followed over the same time period.

$$VE=1-\frac{{\rm{Incidence}}\,{\rm{Rate}}\,{\rm{in}}\,{\rm{Vaccinated}}}{{\rm{Incidence}}\,{\rm{Rate}}\,{\rm{in}}\,{\rm{Unvaccinated}}}$$

For descriptive analyses, the chi-square test for categorical variables and Wilcoxon rank-sum test for continuous variables were used. Annual incidence of BV after vaccination was calculated using life table methods, and stratified by sex and vaccine manufacturer to compare the differences. For the purposes of the study, only the first reported occurrence of breakthrough varicella was included. Every vaccinee was followed from the date of vaccination and censored at the date of the first varicella diagnosis or the end of the study period, December 31, 2014. The Wilcoxon test was applied to check if the difference in incidence was statistically significant in a survival analysis.

The multiple Cox proportional hazard regression model was then used to assess possible risk factors for BV, using the BV event as the outcome. In order to minimize possible herd immunity effects and geographical differences, the calendar year at vaccination and geographical area of residence were used as stratification variables within the model to ensure comparison of children of similar calendar year at vaccination and residential area.

Data management and statistical analysis were performed using SAS and SAS Enterprise Guide software (SAS Institute, Inc, Cary, NC).

## Electronic supplementary material

Supplementary information

## Data Availability

The data that support the findings of this study are available from the National Health Insurance database maintained by the Health and Welfare Data Science Center, Ministry of Health and Welfare, Taiwan. However, restrictions apply to the availability of these data, which were used under license for the current study, and so are not publicly available.
