# Supplementary material for: Epidemiology of Breakthrough Varicella after the Implementation of a Universal Varicella Vaccination Program in Taiwan, 2004–2014
Source: Sci Rep. 2018 Nov 21;8:17192. doi: 10.1038/s41598-018-35451-y (PMC6249209; doi:10.1038/s41598-018-35451-y)
Supplement: Supplementary file 1 — Supplementary information [file 41598_2018_35451_MOESM1_ESM.pdf]

Supplementary information

Epidemiology of Breakthrough Varicella after the Implementation of a Universal Varicella Vaccination Program in Taiwan, 2004–2014

Hao-Yuan Cheng[1, 2#]=, Luan-Yin Chang[2#], Chun-Yi Lu[2], and Li-Min Huang[2]*

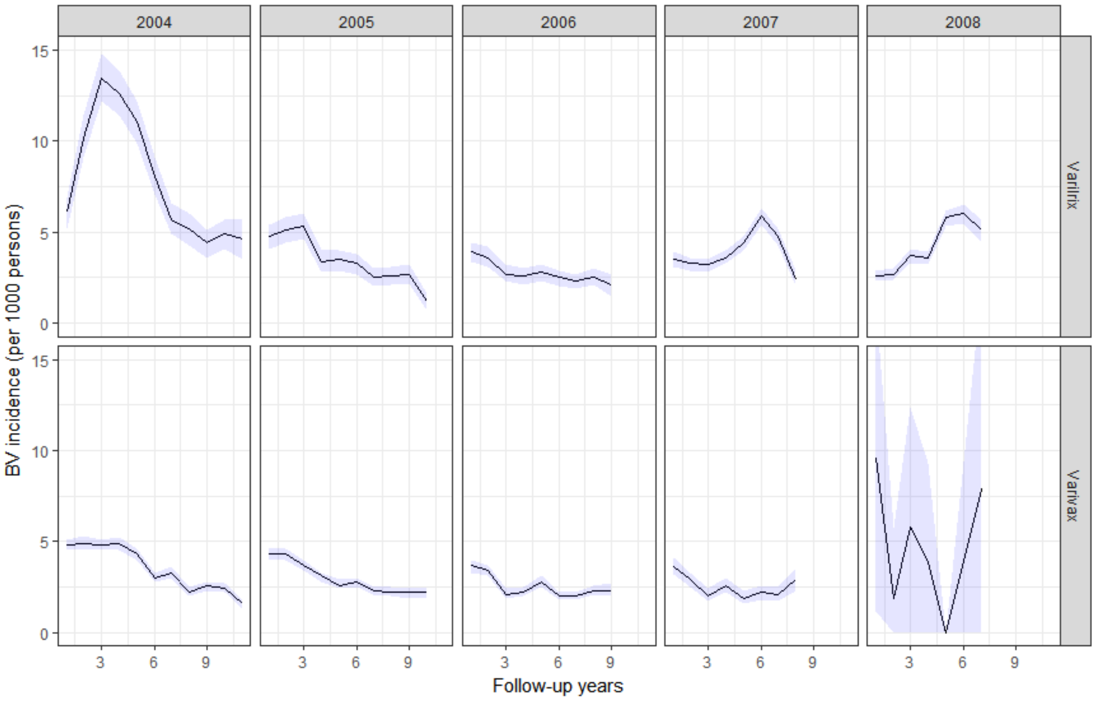

Supplementary Figure S1. Annual BV incidence in all varicella vaccinees by follow-up years, year at vaccination and vaccine manufacturers, 2004–2014. Solid lines represent point estimates, and shadowed lines represent 95% confidence intervals.
